# Trends in Unintentional Drowning Mortality Among U.S. Adults Aged ≥25 Years, 1999–2024: A U.S. Surveillance Analysis

**DOI:** 10.3390/healthcare14070920

**Published:** 2026-04-01

**Authors:** Akef Obeidat, Mohammad Dawar Zahid, Eshal Atif, Sadia Qazi, Anushah Faheem Ilyas, Fnu Urooba, Mazhar Ali, Vishan Das, Muhammad Rai Hassan Ashraf, Muhammad Atif Mazhar

**Affiliations:** 1 Department of Anatomy, College of Medicine, Alfaisal University, Riyadh 11533, Saudi Arabia; aobeidat@alfaisal.edu; 2 Medical College, Agha Khan University, Karachi 74800, Pakistan; mohammaddawar.zahid@scholar.aku.edu; 3 College of Medicine, Alfaisal University, Riyadh 11533, Saudi Arabia; eatif@alfaisal.edu; 4 Department of Medicine, Karachi Medical and Dental College, Karachi 74700, Pakistan; anushah.faheem@gmail.com; 5 Medical College, CMH Institute of Medical Sciences, Multan 53400, Pakistan; uroobatariq91@gmail.com; 6 Khyber Medical College, Peshawar University, Peshawar 25120, Pakistan; mazharaliuss@gmail.com; 7 Medical College, Liaquat University of Medical & Health Sciences, Jamshoro 76090, Pakistan; vishankhoila03@gmail.com; 8 Shifa College of Medicine, Islamabad 44000, Pakistan; rai.hassan.ashraf@gmail.com

**Keywords:** drowning, submersion, mortality, CDC WONDER, joinpoint regression, age-adjusted mortality rate, health disparities, rural health, injury prevention, water safety

## Abstract

**Highlights:**

**What are the main findings?**

- Adult drowning mortality among U.S. adults aged ≥25 years was stable from 1999 to 2013, then rose significantly through 2024 (APC: 1.32%, *p* = 0.012), with the steepest increases among females (AAPC: 1.27%, *p* < 0.001) and adults aged 65–85+ (AAPC: 1.15%, *p* < 0.001); the male-to-female rate ratio narrowed significantly from 4.00 to 3.32 over 25 years (*p* = 0.0006), reflecting divergent sex trajectories.
- Marked disparities persisted throughout the study period: non-Hispanic AI/AN adults carried the highest age-adjusted mortality rates, and non-metropolitan areas consistently recorded higher drowning mortality than metropolitan areas.

**What are the implications of the main findings?**

- Adult drowning mortality requires a prevention-delivery and health-systems response, equitable access to swimming instruction, supervised aquatic environments, and rapid rescue rather than individual-risk messaging alone; the sustained rise in female mortality is an underrecognized signal requiring sex-disaggregated, comorbidity-aware intervention design.
- Persistent racial and geographic disparities reflect structural inequities in aquatic access and emergency response capacity; equity-focused, community-based prevention aligned with U.S. National Water Safety Action Plan implementation is needed, with particular attention to rural rescue readiness and older adult risk embedded in routine geriatric care.

**Abstract:**

Background/Objectives: Drowning is a leading preventable cause of unintentional injury death, yet U.S. prevention efforts have largely focused on children. Despite international declines in pediatric drowning mortality, adult trends remain poorly characterized. We examined long-term trends and disparities in unintentional drowning mortality among U.S. adults aged ≥25 years from 1999 to 2024. Methods: Using CDC WONDER Multiple Cause of Death data, drowning deaths were identified using ICD-10 codes W65–W74, V90, and V92. Age-adjusted mortality rates (AAMRs) per 100,000 were computed by direct standardization to the 2000 U.S. standard population. Joinpoint regression estimated the annual percent change (APC) and average annual percent change (AAPC). Three sensitivity analyses assessed transport-related code exclusion, pandemic-era restriction, and multiple cause-of-death coding. Results: During 1999–2024, 101,743 unintentional drowning deaths occurred among U.S. adults aged ≥25 years (76,554 males; 25,201 females), with 58.09% in natural water or outdoor settings. The overall AAMR showed a non-significant increase (AAPC: 0.55%, *p* = 0.054); however, joinpoint analysis identified stable rates through 2013 followed by a significant sustained increase (APC: 1.32%, 95% CI: 0.32–2.32, *p* = 0.012). The male-to-female rate ratio narrowed significantly from 4.00 (1999) to 3.32 (2024) (ratio of rate ratios: 0.83, *p* = 0.0006), driven by a sustained female increase (AAPC: 1.27%, *p* < 0.001). Adults aged 65–85+ showed the steepest rise (AAPC: 1.15%, *p* < 0.001). Non-Hispanic AI/AN adults had the highest rates (3.47–5.44 per 100,000), and non-metropolitan areas consistently exceeded metropolitan rates. Conclusions: A significant upward trajectory has persisted since 2013, with marked disparities by age, sex, race/ethnicity, and geography. Adult-focused, equity-driven prevention strategies aligned with USNWSAP implementation are needed to address this underrecognized burden.

## 1. Introduction

Drowning is one of the most preventable yet persistently neglected causes of death worldwide. The World Health Organization estimates that more than 300,000 people die from drowning annually, making it the third leading cause of unintentional injury-related death globally [1,2]. Despite a 38% decline in global drowning death rates between 2000 and 2021 [2], drowning continues to claim lives at a rate of approximately 30 people every hour, with over 90% of deaths occurring in low and middle-income countries [1,2]. In the United States, an average of 4083 unintentional drowning deaths occur each year, and approximately 8100 non-fatal drowning injuries are treated in emergency departments annually [3,4]. Drowning is the leading cause of death for children aged 1–4 years and the second leading cause of unintentional injury death for children aged 5–14 years [3,5]. However, the epidemiological profile of adult drowning, which accounts for most drowning fatalities in high-income countries, remains substantially understudied [6,7].

The disproportionate focus on pediatric drowning in research, surveillance, and prevention programs has created critical knowledge gaps regarding adult drowning mortality patterns. A systematic review of adult drowning prevention interventions identified only 22 studies meeting the inclusion criteria over a decade of publication (2011–2021), underscoring the paucity of evidence specific to adult populations [6]. Following evidence linking swim lessons with reduced drowning risk in young children, drowning-prevention messaging has increasingly emphasized early childhood swim instruction, while evidence for adolescents and adults remains comparatively limited [8]. This gap has practical consequences: while childhood drowning death rates in the United States declined by 38% from 1999 to 2019 [9], this decline is part of a broader international pattern, a multi-country analysis of WHO Mortality Database records documented reductions in child and adolescent drowning mortality in 20 of 21 countries during 2000–2013, a period that overlaps precisely with the stable adult drowning rates identified in the present study [10]. The consistency of declining pediatric rates internationally, set against stable or rising adult rates, suggests these represent distinct epidemiological trajectories warranting separate prevention attention. Data from the National Vital Statistics System suggest that adult drowning rates have either plateaued or increased over the same period [11,12]. An NCHS analysis of 1999–2010 data documented that while overall unintentional drowning rates declined by 9%, rates among adults aged 45–84 years increased by 9.7% [11]. A recent study on geriatric drowning found that age-adjusted mortality rates among adults aged ≥ 55 years increased significantly from 1999 to 2020, with an average annual percent change of 1.51% [13].

Racial and ethnic disparities in drowning mortality are both profound and persistent. Non-Hispanic American Indian/Alaska Native (AI/AN) populations experience drowning death rates approximately twice those of non-Hispanic White populations, while non-Hispanic Black populations have rates 1.5 times higher [14,15]. These disparities have not narrowed over two decades of surveillance; the Black-White drowning rate disparity widened from 2005 to 2019 [14]. Disparities in drowning mortality are closely linked to disparities in swimming ability and access to swimming lessons. Approximately 55% of U.S. adults have never taken a formal swimming lesson, with stark differences by race/ethnicity: 63% of Black adults and 72% of Hispanic adults reported never receiving swimming lessons, compared with lower rates among White adults [3]. Nearly 37% of Black adults and approximately 25% of Hispanic adults reported not knowing how to swim [3]. These disparities have deep historical roots in the racial segregation of municipal swimming pools throughout the 20th century, which systematically excluded Black Americans from aquatic facilities and created intergenerational deficits in swimming competency [16,17,18].

The COVID-19 pandemic introduced a disruptive inflection point in drowning mortality trends. A 2024 CDC Vital Signs report documented that over 4500 people died from drowning annually during 2020–2022, approximately 500 more per year than in 2019, reversing decades of decline [3]. The increases were particularly pronounced among young adults aged 20–29 years and among racial and ethnic minority populations. Pandemic-related disruptions, including the closure of public pools, cancelation of swimming lessons, lifeguard shortages, and increased recreational use of natural water bodies, are plausible contributors to this mortality increase, though the ecological nature of available surveillance data precludes causal attribution [3,19]. Powerboat sales reached a 13-year high in 2020, reflecting increased outdoor water recreation that likely occurred without the corresponding safety infrastructure [19].

Current U.S. drowning prevention efforts are coordinated through the National Water Safety Action Plan (USNWSAP), a 10-year roadmap (2023–2032) that represents the first nationally organized drowning prevention strategy in the United States [20]. The USNWSAP includes 99 evidence-informed action recommendations across six domains: barriers, entrapment, and electrical safety; data and public health surveillance; lifeguards and supervision; life jackets and personal flotation devices; rescue and cardiopulmonary resuscitation (CPR); and water safety, water competency, and swimming lessons [20,21]. The plan explicitly acknowledges existing disparities and calls for equity-focused approaches to address them. However, effective implementation requires contemporary granular surveillance data characterizing who is drowning, where, and how these patterns are changing over time.

This study addresses a gap in the literature on drowning mortality by providing a detailed analysis of long-term trends in unintentional drowning mortality among U.S. adults aged ≥25 years from 1999 to 2024. Using joinpoint regression to identify statistically significant trend changes, we examined heterogeneity across age, sex, race/ethnicity, and U.S. Census region and urbanization level. By framing drowning mortality through a health service and prevention delivery lens, we aim to inform the equitable allocation of prevention resources and guide the targeted implementation of the USNWSAP.

## 2. Materials and Methods

### 2.1. Data Source

We obtained mortality data from the Centers for Disease Control and Prevention Wide-ranging Online Data for Epidemiologic Research (CDC WONDER) system, a publicly accessible platform that provides mortality statistics derived from the National Vital Statistics System (NVSS) [22,23]. CDC WONDER aggregates death certificate data from all 50 states and the District of Columbia, enabling population-based analyses of cause-specific mortalities [22,23]. We queried the Multiple Cause of Death database for final mortality data spanning 1999–2024 [23]. The population denominators were derived from the U.S. Census Bureau intercensal and postcensal estimates available through the CDC WONDER.

### 2.2. Case Definition

Unintentional drowning and submersion deaths were identified using International Classification of Diseases, 10th Revision (ICD-10) codes [24]. The primary (underlying-cause) case definition included accidental non-transport drowning and submersion codes W65–W74 and transport-related drowning codes V90 and V92, consistent with CDC drowning surveillance approaches [3,10,13,25]. In the multiple-cause sensitivity analysis, we additionally captured records where drowning/submersion was listed as a contributing condition using T75.1 (drowning and nonfatal submersion) in any record axis [25]. We also repeated analyses excluding V90 and V92 to assess the influence of transport-related drowning on trend estimates.

### 2.3. Study Population

We restricted the analysis to adults aged ≥25 years to separate mature adults from adolescents and young adults (18–24), a group with distinct exposure contexts and risk behaviors that is frequently analyzed separately in drowning surveillance; this improves comparability with prior adult-focused work and reduces age-related heterogeneity [9,10,13].

### 2.4. Variables

Mortality data were stratified by calendar year and by age and broader categories: (25–44 years, 45–64 years, and 65–85+years)**,** sex (male/female), race/ethnicity (non-Hispanic White, non-Hispanic Black, non-Hispanic AI/AN, non-Hispanic API, and Hispanic any race), U.S. Census Bureau region (Northeast, Midwest, South, West) [26], urbanization using the 2013 NCHS Urban–Rural Classification Scheme (large central metro, large fringe metro, medium metro, small metro, micropolitan, noncore) [27], and place of death (home, inpatient facility, outpatient/ED, hospice, nursing home/long-term care, dead on arrival, other, unknown). Race/ethnicity are recorded on death certificates by funeral directors based on next-of-kin report or observation; analyses should be interpreted considering known misclassification, particularly among AI/AN decedents [28,29,30]. Urbanization analyses were limited to years with available NCHS urban–rural coding (1999–2020) only [27].

### 2.5. Statistical Analyses

#### 2.5.1. Age-Adjusted Mortality Rates

Age-adjusted mortality rates (AAMRs) per 100,000 population were calculated using the direct standardization method with the 2000 U.S. standard population as a reference [31,32]. Direct standardization applies age-specific death rates to a standard population age distribution, producing rates that can be compared across populations with different age structures. Ninety-five percent confidence intervals for AAMRs were calculated based on the gamma distribution method, which is appropriate for rates based on small numbers [31,33]. Rates based on fewer than 20 deaths were flagged as statistically unreliable, and rates based on fewer than 10 deaths were suppressed according to the CDC WONDER confidentiality requirements [34,35].

#### 2.5.2. Joinpoint Regression

Temporal trends in AAMRs were analyzed using joinpoint regression implemented with the National Cancer Institute Joinpoint Regression Program (version 5.4.0) [36,37,38]. Joinpoint regression fits a series of joined linear segments to trend data, identifying statistically significant changes in the direction or magnitude of the trends (joinpoint) [36]. This method identifies the minimum number of joinpoints necessary to describe the data using permutation tests at an overall significance level of α = 0.05 [36,39,40]. We specified a maximum of four joinpoints for the overall analysis and three joinpoints for stratified analyses, with a minimum of two observations between joinpoints and from either end of the data. This maximum was selected consistent with NCHS guidelines for long-term mortality trend analysis [39,40]; models allowing higher maximum joinpoint counts were also tested and did not identify additional statistically significant inflection points.

Log-transformed rates were modeled using weighted least-squares regression, with the standard error of the rates as weights [36,39,40]. This approach yields the annual percentage change (APC) as the primary trend measure, representing the estimated percentage change in the AAMR per year within each identified trend segment [36]. The average annual percentage change (AAPC) was calculated as a summary measure of the overall trend across the full study period (1999–2024) as a weighted average of the APCs with weights proportional to the length of each segment [41]. An APC or AAPC was considered statistically significant if the 95% confidence interval excluded zero [41,42]. Given the large number of stratified analyses performed across multiple demographic and geographic strata, the familywise error rate is inflated and some nominally significant subgroup results may represent Type I error; accordingly, effect sizes and 95% confidence intervals are emphasized alongside *p*-values for subgroup comparisons, and borderline findings are interpreted with appropriate caution.

#### 2.5.3. Sensitivity Analyses

We conducted three sensitivity analyses: (1) excluding transport-related drowning codes (V90, V92) to assess their influence on trend estimates; (2) restricting the analysis to 1999–2019 to evaluate trends absent pandemic-related disruptions; and (3) comparing the underlying cause of death with multiple cause-of-death coding approaches to assess potential differences in case ascertainment [43].

### 2.6. Ethical Considerations

This study used publicly available, de-identified mortality data from CDC WONDER and was exempt from institutional review board review per 45 CFR 46.104(d) (4).

## 3. Results

### 3.1. Overall Drowning Mortality Trends and Place of Death

During 1999–2024, 101,743 unintentional drowning and submersion deaths occurred among U.S. adults aged ≥25 years, with an average of approximately 3914 deaths per year. Of these, 76,554 (75.2%) occurred in males and 25,201 (24.8%) in females. The overall age-adjusted mortality rate increased from 1.87 per 100,000 population (95% CI: 1.80–1.93) in 1999 to 1.97 per 100,000 (95% CI: 1.91–2.03) in 2024. Analysis of the place of death revealed that most drowning deaths occurred in the ‘Other’ category (58.09%), which included outdoor and natural water locations. This was followed by deaths at the decedent’s home (18.13%), outpatient/emergency room facilities (13.99%), inpatient facilities (6.60%), dead on arrival (2.41%), unknown locations (0.34%), hospice facilities (0.23%), nursing homes (0.23%), and medical facilities with unknown status (0.02%). See Table S1.

Joinpoint regression analysis identified one significant inflection point in the overall trend. The AAMR remained statistically stable from 1999 to 2013 (APC = −0.05%, 95% CI: −0.76 to 0.66, *p* = 0.878). Beginning in 2013, a statistically significant sustained increase was detected (APC = 1.32%, 95% CI: 0.32 to 2.32, *p* = 0.012), which continued through 2024. The overall average annual percentage change for the full study period was 0.55% (95% CI: −0.01 to 1.11, *p* = 0.054), which did not reach statistical significance at the α = 0.05 level.

### 3.2. Trends by Sex

**Figure 1:**
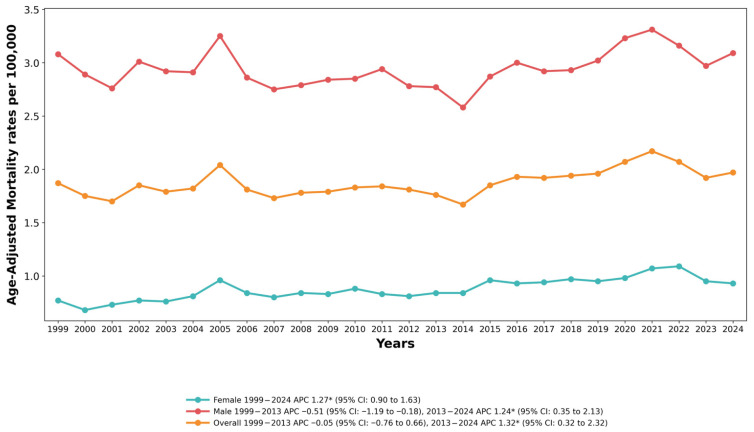
Overall and sex-stratified trends in Drowning and Submersion–related age-adjusted mortality rates per 100,000 among adults in the United States, 1999 to 2024. APC, Annual Percentage Change, CI = Confidence Interval. * Indicates that the Annual Percentage Change (APC) is significantly different from zero at α = 0.05.

Throughout the study period, males experienced substantially higher drowning mortality rates than females. In 1999, the male AAMR was 3.08 per 100,000 (95% CI: 2.96–3.19) compared with 0.77 per 100,000 (95% CI: 0.71–0.82) among females, yielding a formal male-to-female rate ratio of 4.00 (95% CI: 3.69–4.34). By 2024, the male AAMR was 3.09 per 100,000 (95% CI: 2.99–3.20) and the female rate was 0.93 per 100,000 (95% CI: 0.87–0.98), producing a rate ratio of 3.32 (95% CI: 3.10–3.56). The ratio of rate ratios across the study period was 0.83 (95% CI: 0.75–0.92, *p* = 0.0006), indicating a statistically significant narrowing of the male-to-female disparity over 25 years. This narrowing reflects divergent trend trajectories: among females, the AAMR increased significantly and consistently across the entire 25-year period (AAPC: 1.27%, 95% CI: 0.90–1.64, *p* < 0.001) with no joinpoint identified, while among males the overall AAPC was non-significant (0.26%, 95% CI: −0.26 to 0.77, *p* = 0.330), with a period of stability from 1999 to 2013 (APC: −0.51%, 95% CI: −1.19 to 0.18, *p* = 0.140) followed by a significant increase from 2013 to 2024 (APC: 1.24%, 95% CI: 0.35–2.13, *p* = 0.008). The male excess persists but has narrowed substantially, reflecting a sustained female increase against a long-term stable male trajectory. See Figure 1 and Tables S2–S4.

### 3.3. Trends by Age Group

Substantial variations in trend patterns were observed across age groups. The highest AAMRs were recorded in adults aged 65–85+ years, followed by adults aged 45–64 years, with the lowest rates recorded among adults aged 25–44 years.

**Figure 2:**
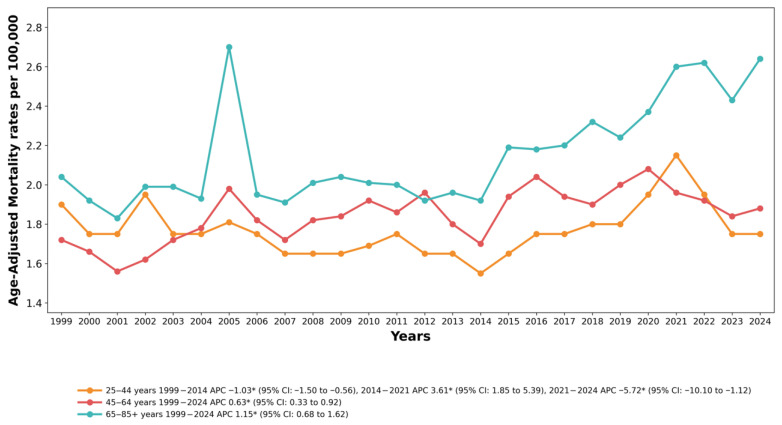
Drowning and Submersion-related Age-adjusted Mortality Rates per 100,000, Stratified by Age-groups in Adults in the United States,1999 to 2024. APC, Annual Percentage Change, CI = Confidence Interval. * Indicates that the Annual Percentage Change (APC) is significantly different from zero at α = 0.05.

Among adults aged 25–44 years, the AAMR declined significantly from 1999 to 2014 (APC = −1.03%, 95% CI: −1.50 to −0.56, *p* < 0.001), followed by a sharp increase through 2021 (APC = 3.61%, 95% CI: 1.85 to 5.39, *p* < 0.001). Subsequently, the rates decreased again through 2024 (APC = −5.72%, 95% CI: −10.11 to −1.13, *p* = 0.018). The overall AAPC for this age group was −0.33% (95% CI: −1.07 to 0.40, *p* = 0.373), which was not statistically significant. See Figure 2 and Table S5.

For adults aged 45–64 years, the AAMR increased significantly and consistently across the study period from 1999 to 2024 (AAPC = 0.63%, 95% CI: 0.33 to 0.92, *p* < 0.001), with no joinpoint identified. This represents a sustained upward trend over the entire 25-year period.

Among adults aged 65–85+ years, the AAMR exhibited a notable and statistically significant increase from 1999 to 2024 (AAPC = 1.15%, 95% CI: 0.68 to 1.62, *p* < 0.001). This age group demonstrated the steepest rate of increase, rising from 2.04 per 100,000 (95% CI: 1.89–2.19) in 1999 to 2.64 per 100,000 (95% CI: 2.51–2.77) in 2024, representing a 29% increase over the study period.

### 3.4. Trends by Race/Ethnicity

Marked disparities in drowning mortality were observed across racial and ethnic groups. Non-Hispanic American Indian/Alaska Native (NH AI/AN) adults had the highest AAMRs of any racial/ethnic group throughout the study period, ranging from 3.47 per 100,000 in 2000 to 5.44 per 100,000 in 2016. Non-Hispanic Black adults had the second highest rates, followed by non-Hispanic White adults. Non-Hispanic Asian/Pacific Islander and Hispanic/Latino adults had the lowest rates.

**Figure 3:**
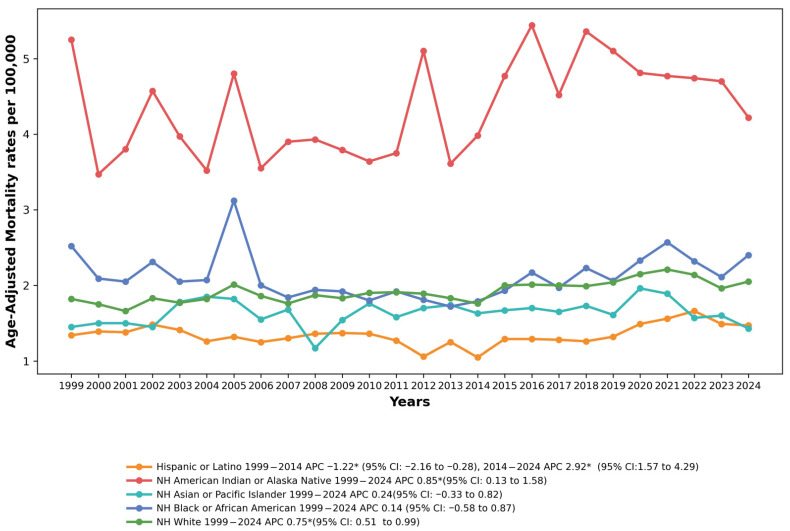
Drowning and Submersion–related Age-Adjusted Mortality Rates per 100,000, Stratified by Race in Adults in the United States,1999 to 2024. APC = Annual Percentage Change, CI = Confidence Interval. * Indicates that the Annual Percentage Change (APC) is significantly different from zero at α = 0.05.

Among NH AI/AN individuals, the joinpoint regression model indicated a statistically significant overall upward trend from 1999 to 2024 (AAPC = 0.85%, 95% CI: 0.13 to 1.58, *p* = 0.023). It should be noted, however, that the absolute AAMR at the endpoints of the study period was 5.25 per 100,000 in 1999 and 4.22 per 100,000 in 2024, reflecting substantial year-to-year variability in this relatively small population. Given the inflated familywise error rate across multiple subgroup comparisons, this borderline-significant finding (*p* = 0.023) should be interpreted as hypothesis-generating; the effect size and confidence interval (AAPC: 0.85%, 95% CI: 0.13–1.58) are more informative than the *p*-value alone. Despite these fluctuations, this population has consistently experienced elevated drowning mortality rates approximately 2–3 times higher than the overall U.S. rate. See Figure 3 and Table S6.

NH White individuals showed a significant increase in AAMR from 1999 to 2024 (AAPC = 0.75%, 95% CI: 0.51 to 0.99, *p* < 0.001), rising from 1.82 per 100,000 in 1999 to 2.05 per 100,000 by 2024.

NH Black or African American individuals demonstrated a non-significant increase over the study period (AAPC = 0.14%, 95% CI: −0.58 to 0.87, *p* = 0.695). The rates ranged from 1.72 per 100,000 in 2013 to 3.12 per 100,000 in 2005, with considerable year-to-year variation.

NH Asian or Pacific Islander individuals showed a non-significant trend (AAPC = 0.24%, 95% CI: −0.33 to 0.82, *p* = 0.392), with rates ranging from 1.17 per 100,000 in 2008 to 1.96 per 100,000 in 2020.

Among Hispanic or Latino individuals, the AAMR declined significantly from 1999 to 2014 (APC = −1.22%, 95% CI: −2.16 to −0.28, *p* = 0.014), followed by a sharp increase from 2014 to 2024 (APC = 2.92%, 95% CI: 1.57 to 4.29, *p* < 0.001). The overall AAPC was 0.41% (95% CI: −0.32 to 1.15, *p* = 0.271).

### 3.5. Trends by Census Region

Mortality trends show disparities across the U.S. Census regions from 1999 to 2024. The South region had the highest AAMRs throughout most of the study period, followed by the West, Midwest, and Northeast regions, respectively.

**Figure 4:**
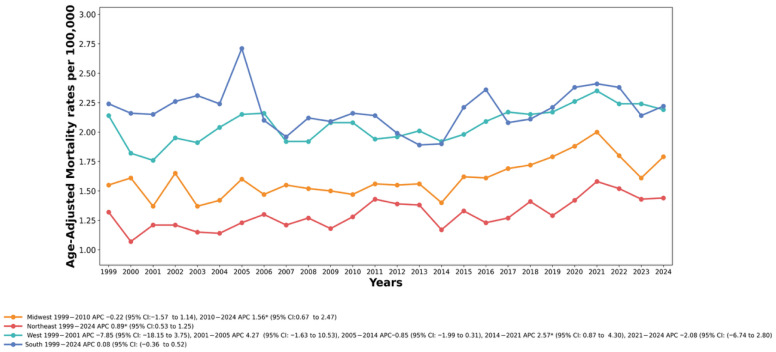
Drowning and Submersion–related Age-Adjusted Mortality Rates per 100,000, Stratified by Census Region in Adults in the United States,1999 to 2024. APC, Annual Percentage Change, CI = Confidence Interval. * Indicates that the Annual Percentage Change (APC) is significantly different from zero at α = 0.05.

In the Northeast region, the AAMR increased significantly from 1999 to 2024 (AAPC = 0.89%, 95% CI: 0.53 to 1.25, *p* < 0.001), rising from 1.32 per 100,000 in 1999 to 1.44 per 100,000 by 2024. See Figure 4; Table S7.

Across the Midwest region, the AAMR remained relatively stable from 1999 to 2010 (APC = −0.22%, 95% CI: −1.57 to 1.14, *p* = 0.736), followed by a notable rise from 2010 to 2024 (APC = 1.56%, 95% CI: 0.67 to 2.47, *p* = 0.002). The overall AAPC for the Midwest was 0.77% (95% CI: 0.04–1.52, *p* = 0.039). Given the inflated familywise error rate, this borderline finding should be interpreted with caution, emphasizing the confidence interval and overall pattern of results rather than relying on the *p*-value alone.

For the southern region, the AAMR remained stable throughout the study period (AAPC = 0.08%, 95% CI: −0.36 to 0.52, *p* = 0.709), with rates fluctuating between 1.89 per 100,000 in 2013 and 2.71 per 100,000 in 2005.

In the western region, the joinpoint analysis identified multiple trend segments. The AAMR remained stable from 1999 to 2014, and then increased significantly between 2014 and 2021 (APC = 2.57%, 95% CI: 0.87 to 4.31, *p* = 0.006), followed by a non-significant decline through 2024 (APC = −2.08%, 95% CI: −6.74 to 2.80, *p* = 0.364). The overall AAPC was 0.17% (95% CI: −1.25 to 1.60, *p* = 0.817).

### 3.6. Trends by Urbanization Level

**Figure 5:**
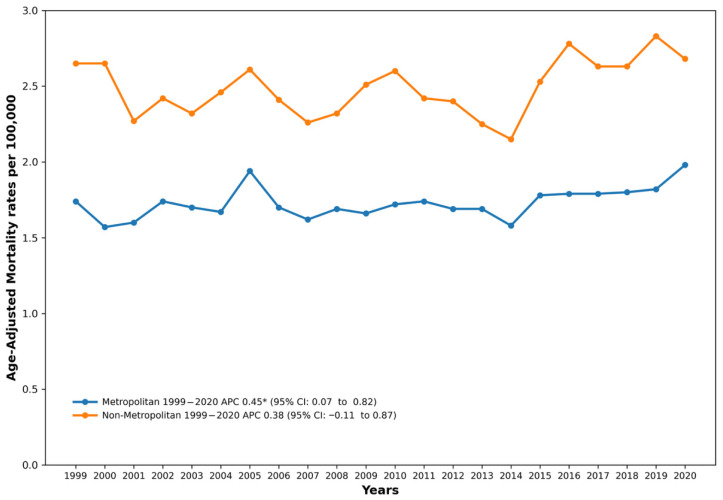
Drowning and Submersion–related Age-Adjusted Mortality Rates per 100,000, Stratified by Urbanization Classification in Adults in the United States,1999 to 2020. APC, Annual Percentage Change, CI = Confidence Interval. * Indicates that the Annual Percentage Change (APC) is significantly different from zero at α = 0.05.

A consistent inverse gradient was observed between urbanization level and drowning mortality throughout the study period. Non-metropolitan counties consistently exhibited higher AAMRs than metropolitan counties. Note: Urbanization data were available only through 2020; therefore, this analysis covers 1999–2020. See Figure 5 and Table S8.

In metropolitan areas, the AAMR increased significantly from 1999 to 2020 (AAPC = 0.45%, 95% CI: 0.07 to 0.82, *p* = 0.022), rising from 1.74 per 100,000 in 1999 to 1.98 per 100,000 by 2020.

In non-metropolitan areas, the rates were consistently higher, ranging from 2.15 per 100,000 in 2014 to 2.83 per 100,000 in 2019. The AAPC from 1999 to 2020 was 0.38% (95% CI: −0.11 to 0.87, *p* = 0.126), which was not statistically significant. The non-metropolitan-to-metropolitan rate ratio ranged from approximately 1.3–1.6 across the study period.

### 3.7. Trends by State

**Figure 6:**
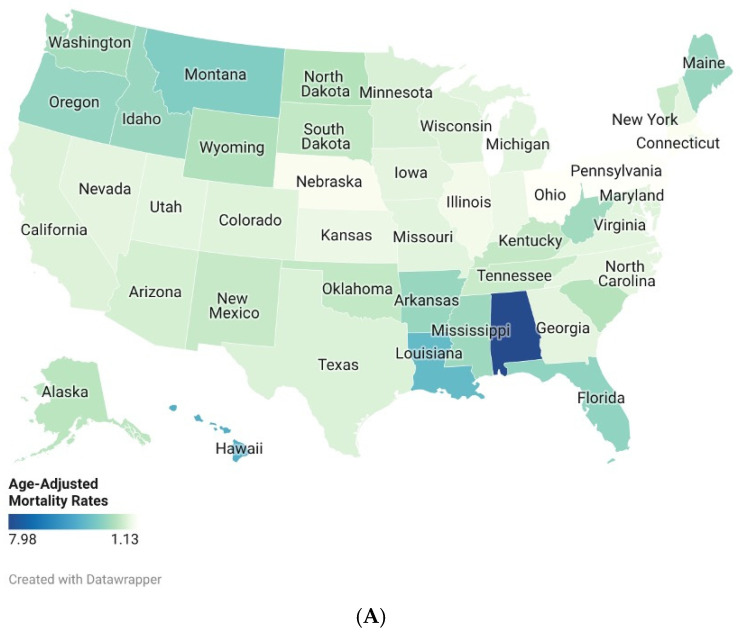

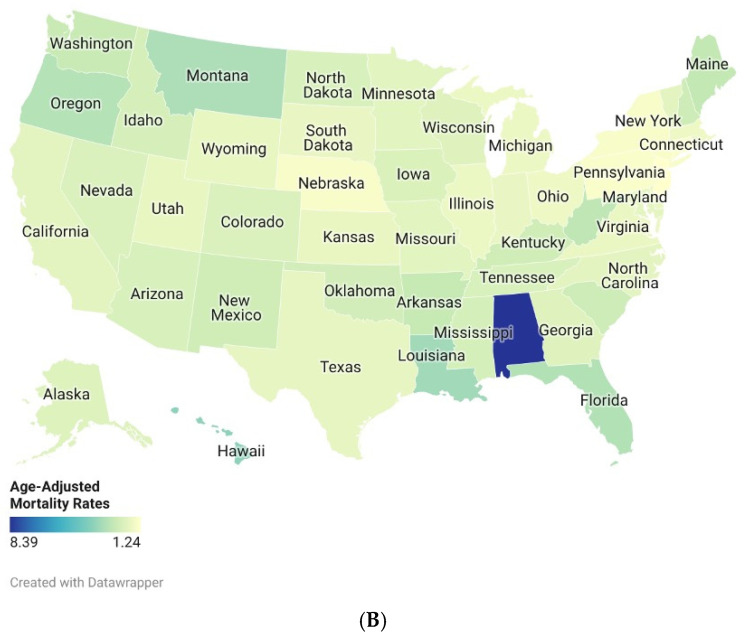
(**A**). Drowning and submersion-related Age-Adjusted Mortality Rates per 100,000, Stratified by States in Adults in the United States, 1999 to 2020. (**B**). Drowning and Submersion-related Age-adjusted Mortality Rates per 100,000, Stratified by States in Adults in the United States, 2021 to 2024.

AAMRs varied substantially across the U.S. states. Analysis from 1999 to 2020 revealed that Alaska had the highest AAMR at 7.98 per 100,000 (95% CI: 7.40–8.57), while New York had the lowest at 1.13 per 100,000 (95% CI: 1.09–1.17). The states in the top 90th percentile included Alaska, Hawaii (4.32 per 100,000), Louisiana (3.89 per 100,000), Montana (3.23 per 100,000), and Florida (2.95 per 100,000). Conversely, the states in the lowest 10th percentile were New York, the District of Columbia (1.22 per 100,000), Pennsylvania (1.22 per 100,000), New Jersey (1.23 per 100,000), and Ohio (1.27 per 100,000). See Figure 6A; Table S9.

From 2021 to 2024, Alaska continued to have the highest AAMR at 8.39 per 100,000 (95% CI: 7.04–9.74), whereas New Jersey recorded the lowest at 1.24 per 100,000 (95% CI: 1.10–1.38). The states in the top 90th percentile included Alaska, Hawaii (3.83 per 100,000), Louisiana (3.47 per 100,000), Montana (3.21 per 100,000), and Florida (3.09 per 100,000). The states in the lowest 10th percentile were New Jersey, Connecticut (1.39 per 100,000), Nebraska (1.45 per 100,000), New York (1.44 per 100,000), and Pennsylvania (1.39 per 100,000). See Figure 6B; Table S9

### 3.8. Results of Sensitivity Analyses

Sensitivity analyses excluding transport-related drowning codes (V90, V92) yielded findings consistent with the main analysis, demonstrating the robustness of the overall trend. Both sexes exhibited rising mortality, although the increase was statistically significant among females (AAPC = 1.26%, 95% CI: 0.90 to 1.63, *p* < 0.001), whereas changes for males remained non-significant (AAPC = 0.25%, 95% CI: −0.25 to 0.77, *p* = 0.329).

Significant increases were observed among NH American Indian or Alaska Native (AAPC = 0.85%, 95% CI: 0.12 to 1.58, *p* = 0.02) and NH White (AAPC = 0.74%, 95% CI: 0.50 to 0.99, *p* < 0.001) populations. Upward trends for NH Black or African American (AAPC = 0.13%, 95% CI: −0.58 to 0.86, *p* = 0.694), NH Asian or Pacific Islander (AAPC = 0.24%, 95% CI: −0.33 to 0.82, *p* = 0.392), and Hispanic or Latino (AAPC = 0.41%, 95% CI: −0.32 to 1.15, *p* = 0.271) populations were not statistically significant.

Regionally, mortality increased significantly in the Northeast (AAPC = 0.88%, 95% CI: 0.52 to 1.24, *p* < 0.001) and Midwest (AAPC = 0.77%, 95% CI: 0.03 to 1.51, *p* = 0.03) regions. The South (AAPC = 0.08%, 95% CI: −0.36 to 0.52, *p* = 0.708) and West (AAPC = 0.16%, 95% CI: −1.24 to 1.60, *p* = 0.817) exhibited upward trends that were not statistically significant.

Both metropolitan and non-metropolitan areas showed upward trends, but the increase reached significance only in metropolitan areas (AAPC = 0.44%, 95% CI: 0.07 to 0.82, *p* = 0.02) compared to non-metropolitan areas (AAPC = 0.37%, 95% CI: −0.11 to 0.87, *p* = 0.125).

These findings confirm that the observed mortality trends persisted when using a more specific case definition, demonstrating the robustness of the main results.

When the analysis was restricted to 1999–2019, excluding pandemic and post-pandemic years, the overall joinpoint model selected zero joinpoints, yielding a single non-significant APC of 0.26% (95% CI: −0.10 to 0.62, *p* = 0.151) across the full pre-pandemic period. The 2013 inflection point identified in the primary analysis was not independently detected within the 1999–2019 window, indicating that the statistical support for the post-2013 segment relies on the slope contrast provided by the 2020–2024 data. Among females, however, the pre-pandemic trend remained statistically significant (APC: 1.21%, 95% CI: 0.73–1.69, *p* < 0.001), consistent with the primary analysis and confirming that the sustained female increase is not a pandemic artifact. Among males, the pre-pandemic trend was non-significant (APC: −0.09%, 95% CI: −0.46 to 0.27, *p* = 0.600).

## 4. Discussion

This national mortality surveillance analysis reveals a concerning epidemiological shift in drowning mortality among U.S. adults aged ≥25 years: a prolonged period of rate stability from 1999 to approximately 2013, followed by a sustained and statistically significant increase continuing through 2024. This finding contrasts with the well-documented decline in pediatric drowning rates over the same period [9] and challenges the assumption that drowning mortality in the United States is decreasing. The heterogeneity of trends across demographic and geographic subgroups points to structural determinants of drowning risk that merit attention through a health services and prevention delivery lens, including where prevention services are available, who can access them, and how quickly rescue and resuscitation can be delivered to the victims.

### 4.1. The Post-2013 Inflection and Key High-Risk Groups

While the overall 25-year AAPC did not reach statistical significance (0.55%, *p* = 0.054), the joinpoint-identified post-2013 increase (APC: 1.32%, *p* = 0.012) represents a clear and significant change in trajectory. This pattern differs from pediatric drowning trends, which have declined in many settings. A multi-country analysis of WHO Mortality Database records documented decreasing child and adolescent drowning mortality in 20 of 21 countries during 2000–2013 [10], the precise period in which adult rates in the present study remained stable. The contrast between these internationally declining pediatric trajectories and the subsequent rise in U.S. adult rates reinforces the interpretation that adult drowning represents a distinct and relatively neglected epidemiological problem [44,45].

The transition from stability to increase around 2013, confirmed by joinpoint regression and robust sensitivity analyses, likely represents a real epidemiological signal rather than solely a COVID-19 artifact. The pre-pandemic sensitivity analysis (1999–2019) did not independently detect the 2013 joinpoint; when the data were truncated at 2019, the software selected zero joinpoints and returned a non-significant overall APC of 0.26% (95% CI: −0.10 to 0.62, *p* = 0.151), indicating that the six-year post-2013 pre-pandemic segment alone did not generate sufficient slope contrast to reach the joinpoint detection threshold. However, among females the pre-pandemic trend remained statistically significant (APC: 1.21%, 95% CI: 0.73–1.69, *p* < 0.001), confirming that the female trajectory is not a pandemic artifact. Among males, the pre-pandemic trend was non-significant (APC: −0.09%, 95% CI: −0.46 to 0.27, *p* = 0.600). The overall post-2013 inflection is therefore best interpreted as a trend that became statistically detectable as the rising trajectory extended through and beyond the pandemic years. The 2024 CDC Vital Signs report documented over 4500 annual drowning deaths during 2020–2022, roughly 500 more per year than in 2019 [3], and these pandemic-era figures appear to have amplified a pre-existing upward trajectory, particularly among females, rather than representing an isolated disruption.

The mechanisms underlying the post-2013 inflection are not directly measurable from death certificate data, but several plausible determinants warrant consideration within a Social Determinants of Health and Haddon matrix framework [46,47]. Demographic aging of the U.S. population increased the absolute size of the older adult cohort most susceptible to drowning. The post-2008 economic recovery coincided with rising participation in outdoor recreational water activities and a documented increase in powerboat registrations, expanding exposure to uncontrolled natural water environments among adults with variable water competency. These represent testable hypotheses, not findings of the present analysis; establishing their relative contribution would require individual-level linked data beyond what death certificate surveillance can provide.

The most striking subgroup findings were the significant long-term increases among females (AAPC: 1.27%) and adults aged ≥65 years (AAPC: 1.15%), both of which showed sustained upward trends across the entire study period. The acceleration of mortality in older adults aligns with the concept of a hidden epidemic in older adult drowning [48], driven by factors including cardiac events, seizure disorders, cognitive impairment, polypharmacy, and declining functional reserve [49,50,51,52]. From a life course epidemiology perspective, these risk factors accumulate with age and interact with environmental exposures, bathtub use, recreational water access, and reduced physiological reserve in ways that static individual-risk framing cannot capture [53]. This finding has direct clinical implications: drowning prevention for older adults should be integrated into routine geriatric care pathways, specifically through medication reconciliation, fall-risk assessment, seizure counseling, and bathing safety education [48,49]. Community-level interventions should include targeted outreach to older adults in high-burden settings, supported by training of primary care providers to identify and counsel patients at elevated drowning risk.

### 4.2. Persistent Disparities and Structural Determinants

The persistent elevation of drowning mortality among non-Hispanic AI/AN and non-Hispanic Black adults is one of the most consequential findings of this analysis. These disparities align with structural determinants of swimming ability, water safety access, and emergency response capacity rather than individual behavior alone. The historical segregation of municipal swimming pools in the United States systematically excluded Black Americans from aquatic facilities throughout the 20th century [16,17], with enduring consequences for aquatic risk documented in subsequent generations [54]. Subsequent disinvestment in public aquatic infrastructure in predominantly Black neighborhoods helped entrench intergenerational deficits in swimming competency [16,17]. National surveillance indicates substantial inequities in swimming skills and formal lesson participation [3,55], and survey-based evidence suggests intergenerational transmission of limited swimming ability within families [56].

Viewed through the lens of Social Determinants of Health, these patterns reflect the downstream consequences of structural inequity in physical infrastructure, economic access, and institutional exclusion determinants that operate across the life course and are not addressable through individual-level behavioral messaging alone. Recent work linking drowning mortality with county-level social vulnerability quantifies this relationship directly: counties with high overall social vulnerability experienced fatal drowning rates 1.59 times those of low-vulnerability counties, with the association strongest for counties with high proportions of mobile homes (RR: 1.62), a proxy for rurality and proximity to unmanaged natural water, and for low socioeconomic status (RR: 1.56) [57].

From a prevention delivery standpoint, swimming instruction functions as a preventive service with unequal access. Structural barriers to swimming participation among minority groups, including cost, transportation, facility access, and social constraints, have been documented [58]. Although the protective association between formal swimming lessons and drowning risk has been demonstrated primarily in pediatric populations [8], emerging evidence suggests that adult swimming instruction is associated with improved water competency [59], and the core principle that access to instruction is consequential and inequitable applies across the lifespan. The finding that 58% of adult drowning deaths in this analysis occurred in “other” locations predominantly natural water bodies, combined with persistently higher rates among AI/AN and Black adults, is consistent with documented disparities in access to supervised aquatic facilities and lifeguarded venues in minority-serving communities. These findings support framing drowning prevention as a health equity and service delivery problem requiring targeted investment in instruction, safe aquatic access, and community-based water safety programming [18,19,60].

The elevated AI/AN drowning mortality rates require interpretive caution because race/ethnicity on death certificates can be misclassified, and misclassification is particularly well documented for AI/AN decedents [28,29,30,61]. Misclassification typically biases AI/AN mortality downward; therefore, the already elevated rates observed in our analysis likely underestimate the true disparity in some regions [29]. The disparities reported here for AI/AN adults should be understood as conservative estimates, the actual mortality gap relative to the overall U.S. rate is probably larger than these data reflect, particularly in regions with known high misclassification rates.

### 4.3. Geographic Gradients and the Rural Mortality Penalty

The consistent inverse relationship between urbanization and drowning mortality mirrors established injury epidemiology patterns, including the well-documented rural injury mortality penalty [61,62,63,64,65]. Several intersecting mechanisms may contribute to this gradient. Rural emergency response is slower on average, and trauma mortality is higher in rural contexts, reflecting system-level constraints in prehospital care, transport distance, and access to definitive care [66,67,68]. This is particularly critical for drowning, where outcomes deteriorate rapidly with longer submersion durations and delayed resuscitation [69]. Rural exposure is also often associated with uncontrolled natural water bodies (lakes, rivers, irrigation channels) without barriers, signage, or lifeguards [11,70,71]. The finding that 58% of adult drowning deaths in this analysis occurred in “other” locations predominantly natural water settings directly supports this mechanism. The majority of adult drowning deaths are occurring in the same uncontrolled environments that are disproportionately accessible in rural and non-metropolitan areas, where barrier infrastructure, lifeguard presence, and emergency response capacity are all structurally limited. It is also worth noting that the rural–urban analysis in this study was limited to 1999–2020 due to NCHS coding availability; the post-2020 years, when pandemic-related shifts in outdoor recreational water use were most pronounced, cannot be characterized by urbanization level, which constrains interpretation of a finding presented as central to the study’s conclusions [27].

### 4.4. Sex Disparities and Divergent Trend Trajectories

Males have consistently accounted for the large majority of adult drowning deaths throughout the study period, a pattern documented globally [1,2,72]. In 1999, the male age-adjusted mortality rate was 3.08 per 100,000 compared with 0.77 per 100,000 among females, yielding a male-to-female rate ratio of 4.00 (95% CI: 3.69–4.34). By 2024, the male rate was 3.09 per 100,000 and the female rate was 0.93 per 100,000, producing a rate ratio of 3.32 (95% CI: 3.10–3.56). The ratio of rate ratios across the study period was 0.83 (95% CI: 0.75–0.92, *p* = 0.0006), indicating a statistically significant narrowing of the male-to-female disparity over 25 years. This narrowing reflects divergent trend trajectories rather than any improvement in male outcomes: female AAMR increased significantly and consistently across the entire study period (AAPC: 1.27%, *p* < 0.001), while the overall male AAPC was non-significant (0.26%, *p* = 0.330), though males showed a significant post-2013 increase (APC: 1.24%, *p* = 0.008) that mirrors the overall population pattern. The sustained female increase, confirmed as non-pandemic in origin by the pre-pandemic sensitivity analysis, represents an epidemiological signal that has been largely absent from drowning prevention discourse historically oriented toward male recreational water exposure [6].The behavioral risk profile differs between sexes in ways that have prevention implications. Males report higher water exposure, greater risk-taking around water, and higher rates of alcohol use during water recreation [72,73]. Alcohol is detected in a substantial fraction of adult drowning deaths, and higher blood alcohol concentrations are associated with sharply increased risk [73,74,75,76]. These factors plausibly contribute to the persistent male excess. The female trajectory, by contrast, may reflect different exposure patterns including bathtub drowning associated with comorbid conditions such as seizure disorders and cardiac events [49,50,51,52] that overlap with the older adult risk profile described above. This is supported by location-specific data showing that while natural water accounts for the majority of adult male drowning deaths, the residential bathtub represents a disproportionately lethal environment for older women, with the proportion of bathtub-related deaths increasing steadily with advancing age for both sexes [77]. Adult drowning prevention requires sex-disaggregated intervention design [20]: targeted alcohol-risk counseling at the point of care and in recreational settings for males, and comorbidity-aware bathing safety programming integrated into routine care for older females. Generic water safety messaging does not address either profile adequately.

The sustained monotonic increase among adults aged 45–64 years (AAPC: 0.63%, *p* < 0.001), with no joinpoint identified across the full 25-year period, deserves separate attention. Unlike the 25–44 group, which showed a rise peaking around 2021 followed by a subsequent decline, or the ≥65 group, where the increase aligns with aging-related comorbidity accumulation, the 45–64 trajectory is flat and uninterrupted. This working-age group may represent a population with increasing recreational water exposure over time, consistent with the post-2008 economic recovery and documented rise in outdoor water recreation [20] combined with insufficient prevention infrastructure targeted at this age range. Future surveillance should monitor whether this monotonic increase continues or mirrors the post-2021 reversal seen in younger adults.

### 4.5. Implications for the U.S. National Water Safety Action Plan

Our findings provide updated surveillance data to support the targeted implementation of the USNWSAP [20,78]. The observed post-2013 increase in adult drowning mortality indicates that preventive investments should extend beyond the traditional pediatric focus and explicitly address adult risk profiles and settings [9]. The marked disparities by race/ethnicity support the USNWSAP’s equity priorities, including scaling affordable swimming and water competency instruction and culturally responsive water-safety education in underserved communities [20,59]. Economic modeling of two established environmental interventions such as four-sided isolation pool fencing and universal life jacket use on watercraft suggests these measures alone could prevent an estimated 348 drowning deaths and avert approximately $4.5 billion in mortality and medical costs annually in the United States [79]. The gap between this estimated preventable burden and current implementation rates underscores the need for accountable, measurable targets within the USNWSAP framework.

The rural–urban gradient underscores the need for prevention designs adapted to natural water environments and for strengthening rescue readiness through bystander training and EMS-aligned implementation in settings where professional response is delayed [70,73]. From a health services perspective, these priorities require cross-sector coordination, clear local delivery responsibilities, and measurable accountability to reduce overall mortality and disparities.

Based on the patterns identified in this analysis, four actionable priorities emerge for USNWSAP implementation. First, adult-focused prevention programming should be explicitly incorporated into USNWSAP funding allocations and program targets, with outcome metrics disaggregated by age group rather than defaulting to a pediatric focus [9]. Second, subsidized swimming and water competency instruction should be scaled in communities with documented low swim lesson participation specifically those with high proportions of Black, Hispanic, and AI/AN residents, using community health worker outreach models to address cost and transportation barriers [57,59]. Evidence suggests adult aquatic skill acquisition requires sustained engagement; fewer than one in ten adults attending swimming lessons could meet basic water competency benchmarks in one cohort study, underscoring the need for program designs that support repeated and progressive participation rather than single-session instruction [58]. Third, rural EMS systems serving counties with high drowning mortality should be prioritized for bystander CPR training programs and rapid-response protocol development, given the documented association between delayed resuscitation and drowning fatality [69,80,81,82,83,84]. Fourth, older adult drowning prevention should be integrated into existing geriatric care infrastructure, annual wellness visits, fall prevention programs, and medication reconciliation workflows as a low-cost, high-reach delivery mechanism for a population whose risk is rising but whose prevention needs are systematically overlooked [48,49]. Future research should evaluate the cost-effectiveness of each of these intervention models in adult populations, where the evidence base remains substantially thinner than in pediatric drowning prevention [6].

### 4.6. Strengths and Limitations

This study has several strengths. It uses a national mortality surveillance source (NVSS via CDC WONDER) with near-complete coverage of U.S. deaths, enabling population-based estimates over a 26-year time horizon (1999–2024). Standardized age-adjustment to the 2000 U.S. population improves comparability over time and across subgroups. Joinpoint regression provides formal identification of statistically significant trend changes and interpretable APC/AAPC estimates. Stratified analyses across age, sex, race/ethnicity, region, and urbanization identify high-burden and rising-risk populations relevant to targeted prevention. Sensitivity analyses support the robustness of the principal findings.

Several limitations should be considered. Death certificate–based analyses are subject to misclassification and incomplete ascertainment and capture only fatal events; non-fatal drowning represents a substantial additional burden not reflected in these data [85]. The assignment of drowning as the underlying cause of death may vary by jurisdiction and circumstance, particularly when comorbid conditions contribute to the fatal event. Additionally, if ICD-10 drowning code fidelity improved over the 25-year study period with a greater proportion of drowning deaths correctly coded as the underlying cause in later years rather than under unspecified external cause codes, this could produce an upward artifact in rates independent of true mortality change [86]. This possibility cannot be excluded from the present analysis; however, the finding that the post-2013 trend persisted across multiple sensitivity analyses, including multiple-cause-of-death coding, reduces the likelihood that it is entirely attributable to coding drift. ICD-10 drowning codes provide limited detail on context and setting, and the absence of data on drowning circumstance including recreational activity type, swimming ability, alcohol involvement, and medical emergency response time limits the ability to identify specific prevention targets or evaluate intervention effectiveness from these data alone. Complementary surveillance systems, including syndromic surveillance approaches, may help address some of these limitations in future analyses [87,88]. The frequent use of unspecified codes reduces the ability to identify specific drowning environments. Race/ethnicity classification on death certificates is imperfect, with well-documented misclassification for AI/AN decedents that likely biases mortality downward [28,29,30]. The disparities reported for AI/AN adults are therefore conservative estimates, and the true burden is likely larger than reflected in these data. The study is ecological and cannot account for individual-level risk factors such as swimming ability, alcohol exposure, comorbidity burden, or rescue timing. Geographic indicators are relatively coarse and do not capture within-region heterogeneity. The large number of stratified analyses performed across demographic and geographic subgroups inflates the familywise error rate; subgroup findings near the conventional significance threshold including the NH AI/AN AAPC (*p* = 0.023) and Midwest AAPC (*p* = 0.039) should be interpreted as hypothesis-generating rather than confirmatory. Finally, the urbanization analysis was limited to 1999–2020 because NCHS urban–rural classification coding in CDC WONDER does not extend beyond 2020 under the 2013 scheme; the 2023 NCHS Urban–Rural Classification revision does not yet provide coverage for years beyond this period, precluding extension of the analysis through the pandemic and post-pandemic years [27]. The rural–urban gradient therefore cannot be characterized for the years with the most pronounced pandemic-related changes in drowning patterns, which represents a meaningful constraint on a finding presented as central to the study’s conclusions.

## 5. Conclusions

This national mortality surveillance analysis identified a significant shift in U.S. adult drowning epidemiology: after more than a decade of relative stability, drowning mortality among adults aged ≥25 years began rising around 2013 and continued to increase through 2024. While the overall 25-year trend did not reach statistical significance, the post-2013 upward trajectory is clear and significant, and the increase is most pronounced in specific high-risk groups, particularly older adults and females. The male-to-female rate ratio narrowed significantly over the study period, driven entirely by a sustained female increase rather than any improvement in male outcomes, a divergence that existing prevention messaging has not adequately addressed. Persistent inequities across racial/ethnic groups and a strong rural–urban gradient complete the picture of a mortality burden shaped by structural determinants rather than individual risk alone. This trajectory stands in contrast to internationally documented declines in pediatric drowning mortality over the same period, reinforcing that adult drowning is a distinct epidemiological problem requiring its own surveillance and prevention infrastructure.

These patterns argue for treating drowning as a prevention delivery and health systems problem, not only as an individual risk problem. Priorities should include scaling equitable access to swimming and water competency instruction in underserved communities, strengthening layered protection in high-risk natural water settings, and improving time-to-rescue through bystander resuscitation training and rural EMS readiness. For older adults, drowning prevention should be integrated into routine geriatric care pathways. Progress will depend on the accountable implementation of the U.S. National Water Safety Action Plan, using surveillance to target the highest-burden communities and track whether prevention investments reduce disparities, not just overall rates.

## Data Availability

The data presented in this study are openly available in the CDC WONDER Multiple Cause of Death database (1999–2024) at [https://wonder.cdc.gov/mcd.html, accessed on 20 December 2025].

## Supplementary Materials

The following supporting information can be downloaded at: https://www.mdpi.com/article/10.3390/healthcare14070920/s1, Table S1. Drowning & Submersion—related Mortality, Stratified by Place of Death, 1999–2024; Table S2. Drowning & Submersion—related Deaths, Stratified by Sex and Race, 1999–2024; Table S3. Sex—Stratified Age—Adjusted Mortality Rates per 100,000, 1999–2024; Table S4. Annual Percent Change (APC) and Average Annual Percent Change (AAPC) of Mortality Rates; Table S5. Age—Adjusted Mortality Rates per 100,000, Stratified by Age Groups; Table S6. Age—Adjusted Mortality Rates per 100,000, Stratified by Race; Table S7. Age—Adjusted Mortality Rates per 100,000, Stratified by Census Region; Table S8. Mortality Rates per 100,000, Stratified by Urbanization (1999–2020); Table S9. Age—Adjusted Mortality Rates per 100,000, Stratified by State.

## List of Abbreviations

AAMR	Age-adjusted mortality rate
AAPC	Average annual percent change
APC	Annual percent change
AI/AN	American Indian/Alaska Native
CDC	Centers for Disease Control and Prevention
CI	Confidence interval
ICD-10	International Classification of Diseases, 10th Revision
ILCOR	International Liaison Committee on Resuscitation
IRB	Institutional Review Board
NCHS	National Center for Health Statistics
NVSS	National Vital Statistics System
USNWSAP	U.S. National Water Safety Action Plan
WHO	World Health Organization
WONDER	Wide-ranging Online Data for Epidemiologic Research
