# Supplementary material for: Trends in Unintentional Drowning Mortality Among U.S. Adults Aged ≥25 Years, 1999–2024: A U.S. Surveillance Analysis
Source: Healthcare (Basel). 2026 Apr 1;14(7):920. doi: 10.3390/healthcare14070920 (PMC13072863; doi:10.3390/healthcare14070920)
Supplement: Supplementary file 1 [file healthcare-14-00920-s001.zip › healthcare-4188239-supplementary.pdf]

Supplementary Tables: Trends in Unintentional Drowning Mortality Among U.S. Adults Aged ≥25 Years, 1999–2024

Table S1. Drowning & Submersion–related Mortality, Stratified by Place of Death, 1999–2024.

Place of Death	Deaths (1999–2020)	Deaths (2021–2024)	Total Deaths (1999–2024)	% of Total
Medical Facility - Inpatient	5,254	1,297	6,551	6.43%
Medical Facility - Outpatient or ER	12,077	2,530	14,607	14.35%
Medical Facility - Dead on Arrival	3,001	224	3,225	3.17%
Medical Facility - Status unknown	26	0	26	0.03%
Decedent's home	12,835	3,924	16,759	16.47%
Hospice facility	124	56	180	0.17%
Nursing home/long term care	266	24	290	0.28%
Other	48,775	10,829	59,604	58.58%
Place of death unknown	485	16	501	0.49%
Total	82,843	18,900	101,743	100.0%

Table S2. Drowning & Submersion–related Deaths, Stratified by Sex and Race, 1999–2024.

Year	Overall	Female	Male	Asian/PI	Black	White	AI/AN	Hispanic
1999	3,394	740	2,654	91	502	2,483	67	251
2000	3,179	682	2,497	107	424	2,343	49	256
2001	3,150	714	2,436	113	427	2,271	52	287
2002	3,417	765	2,652	108	475	2,465	64	305
2003	3,358	768	2,590	133	426	2,438	54	307
2004	3,410	799	2,611	143	446	2,486	50	285
2005	3,946	1,005	2,941	141	641	2,806	63	295
2006	3,533	885	2,648	136	449	2,593	51	304
2007	3,401	808	2,593	153	414	2,454	58	322
2008	3,593	903	2,690	113	438	2,641	60	341
2009	3,645	910	2,735	146	450	2,641	56	352
2010	3,755	945	2,810	178	432	2,714	57	374
2011	3,808	905	2,903	166	465	2,759	60	358
2012	3,753	916	2,837	187	442	2,730	80	314
2013	3,743	934	2,809	202	424	2,685	58	374
2014	3,587	921	2,666	203	447	2,553	63	321
2015	4,114	1,110	3,004	208	498	2,931	79	398

2016	4,291	1,090	3,201	223	574	2,988	86	420
2017	4,245	1,103	3,142	227	524	2,973	78	443
2018	4,326	1,146	3,180	238	606	2,952	92	438
2019	4,433	1,116	3,317	229	567	3,073	89	475
2020	4,762	1,202	3,560	283	651	3,205	86	537
2021	4,908	1,247	3,661	261	695	3,310	72	570
2022	4,803	1,276	3,527	228	650	3,238	75	612
2023	4,470	1,149	3,321	244	583	2,987	73	583
2024	4,719	1,159	3,560	229	680	3,158	65	587
Total	101,743	25,198	76,545	4,690	13,330	71,877	1,737	10,109

Table S3. Sex-Stratified Age-Adjusted Mortality Rates per 100,000, 1999–2024.

Year	Overall (95% CI)	Males (95% CI)	Females (95% CI)
1999	1.87 (1.80–1.93)	3.08 (2.96–3.19)	0.77 (0.71–0.82)
2000	1.75 (1.69–1.82)	2.89 (2.78–3.00)	0.68 (0.63–0.73)
2001	1.70 (1.64–1.76)	2.76 (2.65–2.87)	0.73 (0.67–0.78)
2002	1.85 (1.79–1.91)	3.01 (2.89–3.12)	0.77 (0.72–0.83)
2003	1.79 (1.73–1.85)	2.90 (2.79–3.01)	0.76 (0.70–0.81)
2004	1.80 (1.74–1.86)	2.91 (2.80–3.02)	0.81 (0.76–0.87)
2005	2.04 (1.98–2.11)	3.25 (3.13–3.36)	0.96 (0.90–1.02)
2006	1.81 (1.75–1.87)	2.86 (2.75–2.97)	0.84 (0.78–0.89)
2007	1.73 (1.67–1.79)	2.75 (2.64–2.86)	0.80 (0.75–0.86)
2008	1.78 (1.72–1.84)	2.79 (2.69–2.90)	0.84 (0.79–0.90)
2009	1.79 (1.73–1.85)	2.84 (2.74–2.95)	0.83 (0.77–0.88)
2010	1.83 (1.77–1.89)	2.85 (2.74–2.96)	0.88 (0.83–0.94)
2011	1.84 (1.78–1.89)	2.94 (2.83–3.05)	0.83 (0.78–0.89)
2012	1.81 (1.75–1.87)	2.78 (2.68–2.89)	0.81 (0.75–0.86)
2013	1.76 (1.70–1.82)	2.77 (2.66–2.87)	0.84 (0.78–0.89)
2014	1.67 (1.62–1.73)	2.58 (2.48–2.68)	0.84 (0.78–0.89)
2015	1.85 (1.79–1.91)	2.87 (2.76–2.97)	0.96 (0.90–1.02)
2016	1.93 (1.87–1.99)	3.00 (2.90–3.11)	0.93 (0.88–0.99)
2017	1.90 (1.84–1.96)	2.90 (2.79–3.00)	0.94 (0.88–1.00)
2018	1.94 (1.88–2.00)	2.93 (2.83–3.04)	0.97 (0.91–1.02)
2019	1.96 (1.90–2.01)	3.02 (2.91–3.12)	0.95 (0.89–1.00)
2020	2.07 (2.01–2.14)	3.23 (3.12–3.33)	0.98 (0.93–1.04)
2021	2.17 (2.11–2.24)	3.31 (3.20–3.41)	1.07 (1.01–1.13)
2022	2.07 (2.01–2.13)	3.16 (3.06–3.27)	1.09 (1.03–1.15)
2023	1.92 (1.86–1.97)	2.97 (2.87–3.08)	0.95 (0.90–1.01)
2024	1.97 (1.91–2.03)	3.09 (2.99–3.20)	0.93 (0.87–0.99)

Table S4. Annual Percent Change (APC) and Average Annual Percent Change (AAPC) of Mortality Rates.

Group	Subgroup	Year Interval	APC (95% CI)	P-Value	AAPC (95% CI)
Overall	—	1999–2013	-0.05 (-0.76 to 0.66)	0.878	0.55 (-0.01 to 1.11)
		2013–2024	1.32* (0.32 to 2.32)	0.012	—
Sex	Male	1999–2013	-0.51 (-1.19 to -0.18)	0.140	0.26 (-0.26 to 0.77)
		2013–2024	1.24* (0.35 to 2.13)	0.008	—
	Female	1999–2024	1.27* (0.90 to 1.63)	< .001	1.27* (0.90 to 1.63)
Race	Asian/PI	1999–2024	0.24 (-0.33 to 0.82)	0.392	0.24 (-0.33 to 0.82)
	Black	1999–2024	0.14 (-0.58 to 0.87)	0.695	0.14 (-0.58 to 0.87)
	White	1999–2024	0.75* (0.51 to 0.99)	< .001	0.75* (0.51 to 0.99)
	AI/AN	1999–2024	0.85* (0.13 to 1.58)	0.023	0.85* (0.13 to 1.58)
	Hispanic	1999–2014	-1.22* (-2.16 to -0.28)	0.014	0.41 (-0.32 to 1.15)
		2014–2024	2.92* (1.57 to 4.29)	< .001	—
Urban	Metro	1999–2020	0.45* (0.07 to 0.82)	0.022	0.45* (0.07 to 0.82)
	Non-metro	1999–2020	0.38 (-0.11 to 0.87)	0.126	0.38 (-0.11 to 0.87)
Age	25–44 years	1999–2014	-1.03* (-1.50 to -0.56)	< .001	-0.34 (-1.07 to -0.41)
		2014–2021	3.61* (1.85 to 5.39)	< .001	—
		2021–2024	-5.72* (-10.1 to -1.12)	0.018	—
	45–64 years	1999–2024	0.63* (0.33 to 0.92)	< .001	0.63* (0.33 to 0.92)
	65+ years	1999–2024	1.15* (0.68 to 1.62)	< .001	1.15* (0.68 to 1.62)

Table S5. Age-Adjusted Mortality Rates per 100,000, Stratified by Age Groups.

Year	25–44 years (95% CI)	45–64 years (95% CI)	65+ years (95% CI)
1999	1.90 (1.81–1.99)	1.72 (1.62–1.83)	2.04 (1.89–2.19)
2000	1.75 (1.67–1.84)	1.66 (1.56–1.76)	1.92 (1.77–2.07)
2001	1.75 (1.67–1.84)	1.56 (1.47–1.66)	1.83 (1.69–1.97)
2002	1.95 (1.86–2.05)	1.62 (1.52–1.72)	1.99 (1.85–2.14)
2003	1.75 (1.66–1.84)	1.72 (1.62–1.82)	1.99 (1.85–2.14)
2004	1.75 (1.66–1.84)	1.78 (1.68–1.88)	1.93 (1.79–2.07)
2005	1.81 (1.72–1.90)	1.98 (1.88–2.08)	2.70 (2.53–2.87)
2006	1.75 (1.66–1.84)	1.82 (1.72–1.92)	1.95 (1.81–2.09)
2007	1.65 (1.57–1.74)	1.72 (1.63–1.81)	1.91 (1.77–2.05)
2008	1.65 (1.56–1.73)	1.82 (1.73–1.92)	2.01 (1.87–2.15)
2009	1.65 (1.57–1.74)	1.84 (1.75–1.93)	2.04 (1.90–2.18)
2010	1.69 (1.60–1.78)	1.92 (1.82–2.02)	2.01 (1.87–2.15)
2011	1.75 (1.65–1.84)	1.86 (1.77–1.96)	2.00 (1.87–2.14)
2012	1.65 (1.56–1.73)	1.96 (1.86–2.06)	1.92 (1.79–2.05)
2013	1.65 (1.56–1.73)	1.80 (1.71–1.89)	1.96 (1.83–2.09)
2014	1.55 (1.46–1.63)	1.70 (1.61–1.79)	1.92 (1.79–2.04)
2015	1.65 (1.56–1.73)	1.94 (1.84–2.03)	2.19 (2.05–2.32)

2016	1.75 (1.66–1.83)	2.04 (1.94–2.14)	2.18 (2.05–2.31)
2017	1.75 (1.66–1.84)	1.94 (1.84–2.03)	2.20 (2.07–2.33)
2018	1.80 (1.71–1.89)	1.90 (1.80–2.00)	2.32 (2.19–2.45)
2019	1.80 (1.71–1.89)	2.00 (1.90–2.10)	2.24 (2.11–2.37)
2020	1.95 (1.85–2.04)	2.08 (1.98–2.18)	2.37 (2.24–2.50)
2021	2.15 (2.06–2.25)	1.96 (1.86–2.05)	2.60 (2.46–2.74)
2022	1.95 (1.86–2.05)	1.92 (1.82–2.02)	2.62 (2.49–2.75)
2023	1.75 (1.67–1.84)	1.84 (1.74–1.94)	2.43 (2.30–2.56)
2024	1.75 (1.67–1.85)	1.88 (1.78–1.98)	2.64 (2.51–2.78)

Table S6. Age-Adjusted Mortality Rates per 100,000, Stratified by Race.

Year	Asian/PI	Black	White	AI/AN	Hispanic
1999	1.45 (1.14–1.81)	2.52 (2.29–2.74)	1.82 (1.74–1.89)	5.25 (4.02–6.75)	1.34 (1.15–1.52)
2000	1.50 (1.20–1.81)	2.09 (1.89–2.29)	1.75 (1.68–1.83)	3.47 (2.56–4.59)	1.39 (1.20–1.58)
2001	1.50 (1.21–1.80)	2.05 (1.85–2.24)	1.66 (1.59–1.73)	3.80 (2.81–5.02)	1.38 (1.20–1.56)
2002	1.45 (1.16–1.74)	2.31 (2.10–2.52)	1.83 (1.75–1.90)	4.57 (3.50–5.87)	1.48 (1.30–1.67)
2003	1.78 (1.45–2.10)	2.05 (1.85–2.24)	1.77 (1.70–1.84)	3.97 (2.95–5.22)	1.41 (1.24–1.59)
2004	1.85 (1.53–2.17)	2.07 (1.88–2.27)	1.82 (1.74–1.89)	3.52 (2.60–4.67)	1.26 (1.10–1.42)
2005	1.82 (1.50–2.14)	3.12 (2.87–3.36)	2.01 (1.93–2.09)	4.80 (3.64–6.20)	1.32 (1.15–1.48)
2006	1.55 (1.28–1.82)	2.00 (1.82–2.19)	1.86 (1.79–1.93)	3.55 (2.63–4.70)	1.25 (1.09–1.40)
2007	1.68 (1.40–1.95)	1.84 (1.66–2.02)	1.76 (1.69–1.84)	3.90 (2.94–5.06)	1.30 (1.15–1.46)
2008	1.17 (0.94–1.39)	1.94 (1.75–2.12)	1.87 (1.80–1.94)	3.93 (2.99–5.07)	1.36 (1.20–1.52)
2009	1.54 (1.28–1.80)	1.92 (1.74–2.10)	1.83 (1.76–1.91)	3.79 (2.84–4.96)	1.37 (1.22–1.52)
2010	1.76 (1.50–2.03)	1.80 (1.63–1.97)	1.90 (1.83–1.97)	3.64 (2.74–4.75)	1.36 (1.21–1.51)
2011	1.58 (1.33–1.82)	1.92 (1.74–2.09)	1.91 (1.84–1.99)	3.75 (2.86–4.84)	1.27 (1.13–1.41)
2012	1.70 (1.45–1.95)	1.81 (1.64–1.98)	1.89 (1.82–1.97)	5.10 (4.03–6.36)	1.06 (0.94–1.19)
2013	1.74 (1.49–1.98)	1.72 (1.55–1.89)	1.83 (1.76–1.90)	3.61 (2.72–4.70)	1.25 (1.11–1.38)
2014	1.63 (1.41–1.86)	1.79 (1.62–1.96)	1.76 (1.69–1.84)	3.98 (3.04–5.12)	1.05 (0.93–1.17)

2015	1.67 (1.44–1.91)	1.93 (1.75–2.10)	2.00 (1.93–2.08)	4.77 (3.76–5.97)	1.29 (1.15–1.42)
2016	1.70 (1.48–1.93)	2.17 (1.99–2.35)	2.01 (1.94–2.09)	5.44 (4.33–6.75)	1.29 (1.16–1.42)
2017	1.65 (1.43–1.87)	1.97 (1.80–2.14)	2.00 (1.92–2.08)	4.52 (3.55–5.67)	1.28 (1.16–1.40)
2018	1.73 (1.51–1.95)	2.23 (2.05–2.41)	1.99 (1.92–2.07)	5.36 (4.30–6.60)	1.26 (1.14–1.39)
2019	1.61 (1.40–1.82)	2.06 (1.89–2.23)	2.04 (1.97–2.12)	5.10 (4.08–6.31)	1.32 (1.19–1.44)
2020	1.96 (1.73–2.19)	2.33 (2.15–2.52)	2.15 (2.07–2.23)	4.81 (3.82–5.97)	1.49 (1.36–1.62)
2021	1.89 (1.66–2.12)	2.57 (2.37–2.76)	2.21 (2.13–2.29)	4.77 (3.71–6.04)	1.56 (1.43–1.69)
2022	1.57 (1.37–1.78)	2.32 (2.14–2.50)	2.14 (2.06–2.22)	4.74 (3.71–5.97)	1.66 (1.53–1.79)
2023	1.60 (1.40–1.80)	2.11 (1.93–2.28)	1.96 (1.89–2.04)	4.70 (3.67–5.94)	1.49 (1.37–1.61)
2024	1.43 (1.25–1.63)	2.36 (2.18–2.55)	2.03 (1.96–2.11)	4.22 (3.24–5.41)	1.47 (1.47–1.36)

Table S7. Age-Adjusted Mortality Rates per 100,000, Stratified by Census Region.

Year	Northeast (95% CI)	Midwest (95% CI)	South (95% CI)	West (95% CI)
1999	1.32 (1.20–1.44)	1.55 (1.43–1.67)	2.24 (2.13–2.36)	2.14 (1.99–2.28)
2000	1.07 (0.96–1.18)	1.61 (1.49–1.73)	2.16 (2.04–2.27)	1.82 (1.69–1.96)
2001	1.21 (1.09–1.32)	1.37 (1.26–1.48)	2.15 (2.04–2.26)	1.76 (1.63–1.89)
2002	1.21 (1.10–1.33)	1.65 (1.53–1.77)	2.26 (2.14–2.37)	1.95 (1.81–2.08)
2003	1.15 (1.04–1.26)	1.37 (1.26–1.48)	2.31 (2.19–2.42)	1.91 (1.77–2.04)
2004	1.14 (1.03–1.25)	1.42 (1.30–1.53)	2.24 (2.13–2.35)	2.04 (1.90–2.18)
2005	1.23 (1.12–1.35)	1.60 (1.48–1.72)	2.71 (2.59–2.84)	2.15 (2.01–2.29)
2006	1.30 (1.18–1.42)	1.47 (1.36–1.59)	2.10 (1.99–2.20)	2.16 (2.02–2.29)
2007	1.21 (1.10–1.32)	1.55 (1.43–1.67)	1.96 (1.86–2.07)	1.92 (1.79–2.05)
2008	1.27 (1.15–1.38)	1.52 (1.40–1.64)	2.12 (2.02–2.23)	1.92 (1.79–2.05)
2009	1.18 (1.07–1.29)	1.50 (1.38–1.61)	2.09 (1.98–2.19)	2.08 (1.95–2.22)
2010	1.28 (1.16–1.39)	1.47 (1.36–1.58)	2.16 (2.06–2.27)	2.08 (1.94–2.21)
2011	1.43 (1.31–1.56)	1.56 (1.44–1.67)	2.14 (2.04–2.25)	1.94 (1.81–2.06)
2012	1.39 (1.27–1.50)	1.55 (1.44–1.67)	1.99 (1.89–2.09)	1.96 (1.84–2.09)
2013	1.38 (1.26–1.49)	1.56 (1.44–1.68)	1.89 (1.79–1.98)	2.01 (1.88–2.14)
2014	1.17 (1.06–1.28)	1.40 (1.29–1.51)	1.90 (1.80–1.99)	1.92 (1.80–2.04)
2015	1.33 (1.21–1.45)	1.62 (1.50–1.74)	2.21 (2.11–2.32)	1.98 (1.85–2.10)
2016	1.23 (1.12–1.34)	1.61 (1.49–1.73)	2.36 (2.25–2.46)	2.09 (1.97–2.22)
2017	1.27 (1.16–1.38)	1.69 (1.57–1.82)	2.08 (1.98–2.18)	2.17 (2.04–2.29)
2018	1.41 (1.29–1.54)	1.72 (1.60–1.84)	2.11 (2.01–2.21)	2.15 (2.02–2.28)
2019	1.29 (1.18–1.41)	1.79 (1.67–1.91)	2.21 (2.11–2.31)	2.17 (2.04–2.30)
2020	1.42 (1.30–1.54)	1.88 (1.75–2.01)	2.38 (2.27–2.48)	2.26 (2.13–2.39)

2021	1.58 (1.45–1.71)	2.00 (1.87–2.13)	2.41 (2.30–2.52)	2.35 (2.22–2.48)
2022	1.52 (1.40–1.65)	1.80 (1.68–1.93)	2.38 (2.28–2.49)	2.24 (2.11–2.36)
2023	1.43 (1.31–1.55)	1.61 (1.49–1.73)	2.14 (2.04–2.23)	2.24 (2.11–2.37)
2024	1.44 (1.32–1.56)	1.79 (1.67–1.91)	2.22 (2.12–2.32)	2.19 (2.06–2.31)

Table S8. Mortality Rates per 100,000, Stratified by Urbanization (1999–2020).

Year	Metropolitan (95% CI)	Non-Metropolitan (95% CI)
1999	1.74 (1.67–1.81)	2.65 (2.46–2.84)
2000	1.57 (1.51–1.64)	2.65 (2.46–2.84)
2001	1.60 (1.54–1.67)	2.27 (2.09–2.44)
2002	1.74 (1.68–1.81)	2.42 (2.24–2.60)
2003	1.70 (1.64–1.77)	2.32 (2.14–2.50)
2004	1.67 (1.61–1.74)	2.46 (2.28–2.65)
2005	1.94 (1.87–2.01)	2.61 (2.42–2.80)
2006	1.70 (1.64–1.77)	2.41 (2.23–2.59)
2007	1.62 (1.56–1.68)	2.26 (2.09–2.43)
2008	1.69 (1.63–1.75)	2.32 (2.14–2.49)
2009	1.66 (1.60–1.72)	2.51 (2.33–2.69)
2010	1.72 (1.65–1.78)	2.60 (2.41–2.79)
2011	1.74 (1.68–1.80)	2.42 (2.24–2.60)
2012	1.69 (1.63–1.75)	2.40 (2.22–2.58)
2013	1.69 (1.63–1.75)	2.25 (2.08–2.43)
2014	1.58 (1.52–1.63)	2.15 (1.98–2.32)
2015	1.78 (1.72–1.85)	2.53 (2.34–2.71)
2016	1.79 (1.72–1.85)	2.78 (2.59–2.98)
2017	1.79 (1.72–1.85)	2.63 (2.44–2.81)
2018	1.80 (1.74–1.86)	2.63 (2.44–2.82)
2019	1.82 (1.76–1.88)	2.83 (2.63–3.03)
2020	1.98 (1.92–2.04)	2.68 (2.49–2.87)

Table S9. Age-Adjusted Mortality Rates per 100,000, Stratified by State.

State	1999–2020 Rate (95% CI)	2021–2024 Rate (95% CI)	Trend
Alabama	2.20 (2.09–2.31)	2.08 (1.83–2.33)	↓
Alaska	7.98 (7.40–8.57)	8.39 (7.04–9.74)	↑
Arizona	1.87 (1.78–1.96)	2.24 (2.03–2.45)	↑
Arkansas	2.83 (2.66–3.00)	2.67 (2.31–3.04)	↓
California	1.74 (1.71–1.78)	1.96 (1.87–2.04)	↑
Colorado	1.73 (1.63–1.82)	2.20 (1.97–2.43)	↑
Connecticut	1.32 (1.22–1.42)	1.39 (1.15–1.63)	↑
Delaware	1.75 (1.52–1.98)	1.79 (1.31–2.38)	↑
District of Columbia	1.22 (0.99–1.46)	1.53 (1.02–2.21)	↑
Florida	2.95 (2.89–3.01)	3.09 (2.95–3.23)	↑
Georgia	1.67 (1.60–1.74)	2.06 (1.89–2.23)	↑

Hawaii	4.32 (4.03–4.62)	3.83 (3.14–4.52)	↓
Idaho	2.78 (2.56–3.01)	2.28 (1.86–2.69)	↓
Illinois	1.47 (1.42–1.53)	1.72 (1.58–1.86)	↑
Indiana	1.56 (1.48–1.64)	1.80 (1.59–2.00)	↑
Iowa	1.67 (1.55–1.79)	2.16 (1.84–2.49)	↑
Kansas	1.56 (1.44–1.69)	1.81 (1.49–2.12)	↑
Kentucky	2.09 (1.98–2.21)	2.44 (2.16–2.72)	↑
Louisiana	3.89 (3.73–4.04)	3.47 (3.13–3.81)	↓
Maine	2.83 (2.59–3.07)	2.73 (2.18–3.27)	↓
Maryland	1.73 (1.64–1.82)	1.91 (1.70–2.12)	↑
Massachusetts	1.37 (1.30–1.44)	1.67 (1.49–1.86)	↑
Michigan	1.68 (1.62–1.75)	1.73 (1.57–1.89)	↑
Minnesota	1.81 (1.71–1.90)	1.98 (1.76–2.21)	↑
Mississippi	2.74 (2.58–2.90)	2.31 (1.96–2.66)	↓
Missouri	1.69 (1.61–1.78)	1.98 (1.76–2.19)	↑
Montana	3.23 (2.93–3.53)	3.21 (2.57–3.85)	↓
Nebraska	1.39 (1.24–1.54)	1.45 (1.13–1.82)	↑
Nevada	1.67 (1.54–1.80)	2.20 (1.89–2.52)	↑
New Hampshire	1.64 (1.46–1.83)	2.49 (1.97–3.00)	↑
New Jersey	1.23 (1.17–1.29)	1.24 (1.10–1.38)	↑
New Mexico	2.05 (1.88–2.22)	2.44 (2.01–2.87)	↑
New York	1.13 (1.09–1.17)	1.44 (1.34–1.54)	↑
North Carolina	1.64 (1.57–1.70)	1.94 (1.78–2.11)	↑
North Dakota	2.31 (2.00–2.62)	2.23 (1.62–3.01)	↓
Ohio	1.27 (1.21–1.32)	1.69 (1.54–1.84)	↑
Oklahoma	2.10 (1.98–2.23)	2.39 (2.08–2.69)	↑
Oregon	2.88 (2.74–3.02)	3.05 (2.73–3.38)	↑
Pennsylvania	1.22 (1.17–1.27)	1.39 (1.27–1.52)	↑
Rhode Island	1.74 (1.54–1.95)	1.91 (1.44–2.49)	↑
South Carolina	2.28 (2.16–2.40)	2.49 (2.23–2.76)	↑
South Dakota	2.10 (1.83–2.37)	1.80 (1.29–2.45)	↓
Tennessee	2.01 (1.92–2.10)	2.24 (2.03–2.46)	↑
Texas	1.78 (1.73–1.82)	1.88 (1.79–1.98)	↑
Utah	1.66 (1.52–1.80)	1.84 (1.54–2.14)	↑
Vermont	2.03 (1.73–2.33)	1.97 (1.37–2.74)	↓
Virginia	1.66 (1.58–1.73)	1.76 (1.58–1.93)	↑
Washington	2.57 (2.47–2.67)	2.61 (2.39–2.83)	↑
West Virginia	2.67 (2.47–2.87)	2.83 (2.33–3.32)	↑
Wisconsin	1.76 (1.66–1.85)	2.06 (1.83–2.29)	↑
Wyoming	2.36 (2.02–2.70)	1.82 (1.23–2.60)	↓

*Note: * indicates APC is significantly different from zero ($p < 0.05$). NH = non-Hispanic; PI = Pacific Islander; AI/AN = American Indian/Alaska Native. ↓= decrease, ↑=increase*
